# Coenzyme Q10, Zinc and MDA levels in verruca vulgaris

**DOI:** 10.3906/sag-1909-12

**Published:** 2020-08-26

**Authors:** Selma KORKMAZ, Fevziye Burcu ŞİRİN, İjlal ERTURAN, Halil İbrahim BÜYÜKBAYRAM, Mehmet YILDIRIM

**Affiliations:** 1 Department of Dermatology, Süleyman Demirel University Faculty of Medicine, Isparta Turkey; 2 Department of Biochemistry, Süleyman Demirel University Faculty of Medicine, Isparta Turkey

**Keywords:** Coenzyme Q10, verruca, zinc, MDA

## Abstract

**Background/aim:**

Verruca vulgaris is a benign disease characterized with papillomas on the skin and mucosa. The aim of this study was to investigate the serum levels of coenzyme Q10, MDA, and zinc as well as the lipid profile of verruca vulgaris patients and examine the relationship between these parameters and clinical manifestations of the disease.

**Materials and methods:**

The study included 49 verruca vulgaris patients (mean age: 32.01 ± 14.20 years; 22 males, 27 females) and 40 healthy volunteers (mean age: 31.63 ± 8.98 years; 21 males and 19 females). Coenzyme Q10 levels were assessed by using an enzyme-linked immunosorbent assay. Serum MDA levels were measured spectrophotometrically. Zinc levels were measured using a Perkin Elmer AAnalyst 800 atomic absorption spectrometer with a deuterium background correction and additional standard techniques.

**Results:**

The coenzyme Q10 levels were found to be higher in the verruca vulgaris group compared to the healthy volunteers. However, this increase was not statistically significant (P = 0.195). Zinc levels were significantly lower in the verruca vulgaris group compared to the healthy volunteers (P = 0.002). In the patient group, MDA levels and HDL levels were significantly higher compared to the healthy volunteers (P = 0.023 and P = 0.004, respectively). Additionally, there was no statistically significant difference between the groups in the CoQ10/Total cholesterol ratio (P = 0.433).

**Conclusion:**

Reduced serum zinc levels and increase of oxidative stress in verruca vulgaris may be a factor responsible for development of verruca vulgaris.

## 1. Introduction

Verruca vulgaris is a benign disease characterized with papillomas on the skin and mucosa that is caused by infection with the human papilloma virus (HPV). The warts can be transmitted by direct contact with people or through use of common items with an infected individual. Various factors such as the strength of the skin barrier, viral titre and immunological responses of the patients play a role in the infection. The virus infects the epithelial basal cells and this situation triggers proliferation of epithelial basal cells [1,2]. Although various treatment options for verruca exist, it is occasionally resistant to treatment or may resolve spontaneously due to a natural immune response. Long-term persistence, multiple treatment necessities and recurrence after treatment of the verruca lesions generally suggest the presence of some immunological impairment in virus infected individuals. 

Zinc is an essential element and plays an important role in many basic physiological functions in the body, particularly in the function of the adaptive and innate immune systems [3]. Zinc deficiency can lead to a suppression of the immune response, decrease in the number of T, B and natural (NK) cells and reduced chemotactic responses of neutrophils, monocytes and macrophages. Thus, zinc deficiency may be associated with a tendency to enhance bacterial, fungal and viral infections [4].

Various studies have shown that oxidative stress increases in patients with verruca vulgaris. Reactive oxygen species (ROS) that are generated during oxidative stress may lead to the formation of toxic oxidation products from lipids such as malondialdehyde (MDA), which may play a role in the etiopathogenesis of verruca vulgaris and have an effect on the chronicity of the disease [5]. Coenzyme Q10 is a compound of the ubiquinone family that is synthesized in humans and in all animals. It prevents the onset of lipid peroxidation and damage of biomolecules by interfering with ROS formation. Various other functions of coenzyme Q10 include stabilization of the cell membrane, regulation of cell signalling, gene expression, cell proliferation and apoptosis [6]. Coenzyme Q10 has gained popularity as a dietary supplement in recent years [7,8].

The aim of this study was to investigate the serum levels of coenzyme Q10, MDA, and zinc as well as the lipid profile of verruca vulgaris patients and examine the relationship between these parameters and clinical manifestations of the disease.

## 2. Materials and methods

The ethical approval for this study was obtained from Süleyman Demirel University, Faculty of Medicine Ethics Committee (Number: 119). Informed consent was obtained from all participants before the study was commenced.

### 2.1. Selection of cases for the study

The study included 49 verruca vulgaris patients (mean age: 32.01 ± 14.20 years; 22 males, 27 females) and 40 healthy volunteers (mean age: 31.63 ± 8.98 years; 21 males, 19 females) from the Süleyman Demirel University Research Hospital, Department of Dermatology and Venereology. Verruca vulgaris was diagnosed with clinical assessment. Patients with dermatological diseases other than verruca vulgaris, cigarette/alcohol use, systemic disease such as cardiac, renal or hepatic diseases, diabetes mellitus, infectious diseases, malignancy, inflammatory rheumatoid diseases, pregnancy, or any history of medication within the last 3 months were excluded from the study. The sex, age, locations of lesions, treatment history, and disease duration of the patients were recorded. Dermatological examination was performed by a single dermatologist.

### 2.2. Biochemical tests

Venous blood samples were collected from patients and controlled after an overnight fasting period and were centrifuged at 3000 rpm for 10 min. The serum was separated into eppendorf tubes for analysis and kept at –80 °C until analysis. Coenzyme Q10 levels were assessed by using an enzyme-linked immunosorbent assay kit (Human CoQ10-ELISA kit/Shanghai Sunred Biological Technology Co, Ltd, Shanghai, China) and were expressed in ng/mL. Total cholesterol levels were measured by an enzymatic method on a Beckman Coulter AU5800 (USA) clinical chemistry analyser and expressed in mg/dL. Serum MDA levels were measured spectrophotometrically according to the double heating method of Draper and Hadley and expressed in µmol/L. Zinc levels were measured using a Perkin Elmer AAnalyst 800 atomic absorption spectrometer (USA) with a deuterium background correction and additional standard techniques [9,10]. Zinc levels were expressed in µg/dL.

### 2.3. Statistical analysis

All data in the study were analysed with SPSS for Windows Version 22.0 (IBM Corp., Armonk, NY, USA). Normality of variables was tested with the Kolmogorov–Smirnov test. Descriptive analyses are presented as mean ± standard deviation (SD) or median (minimum-maximum). The chi-square test was performed for qualitative variables. Student’s t-test was used to compare normally distributed variables and Mann–Whitney U test was used for nonnormal distributed variables. Spearman test was used for calculating correlation coefficients and their significance. A P value of <0.05 was considered as significant.

## 3. Results 

There were no statistically significant differences between the patient and control groups in terms of age and sex (P > 0.05 for all comparisons). Descriptive variables, clinical and laboratory characteristics of the study groups are shown in Table 1. The mean duration of verruca vulgaris in the patients ranged from 1–120 months (19.35 ± 28.82). The lesion was located at the genital area in 18 patients (36.7%) and in a nongenital area (hand, foot, face, trunk) in 31 patients (63.3%). The coenzyme Q10 levels were found to be higher in the verruca vulgaris group compared to the healthy volunteers. However, this increase was not statistically significant (P = 0.195). Zinc levels were significantly lower in the verruca vulgaris group compared to the healthy volunteers (P = 0.002). In the patient group, MDA levels and HDL levels were significantly higher compared to the healthy volunteers (P = 0.023 and P = 0.004, respectively). There was no statistically significant difference between the groups in serum total cholesterol, triglyceride, LDL, VLDL levels (P > 0.05 for all). Additionally, there was no statistically significant difference between the groups in the CoQ10/total cholesterol ratio (P = 0.433). Thirty-eight of the patients (77.6 %) were treated with cryotherapy and the remaining 6 (12.2%) were provided with other treatment options (Table 1).

**Table 1:** Descriptives, clinical and laboratory characteristics of the cases and controls.

	Cases (n: 49)	Controls (n: 40)	P
Sex Female, n (%) Male, n (%)	27 (55.1 %) 22 (44.9 %)	19 (47.5%) 21 (52.5%)	0.475*
Age	32.01 ± 14.20, 26.00 (18–65)	31.63 ± 8.98, 32.00 (20–53)	0.866** 0.270***
Duration of verruca, month, mean ± SD	19.35 ± 28.82 (1–120)	-	
Patients with genital verruca, n(%)	18 (36.7)	-	
Patients with nongenital (hand, foot, face, trunk) verruca,n (%)	31 (63.3)	-	
Number of verrucas, mean ± SD (min–max)	9.22 ± 14.88 (1–100)	-	
Treatment Cryotherapy, n (%) Others (imiquimod, salicylic acid, and 5-fluorouracil) n (%)	38 (77.6 %) 6 (12.2 %)		
Zinc, median (min–max)	77.73 (52.5–113.9)	91.50 (57.4–215.9)	0.002***
Co enzyme Q, median (min–max)	35.98 (8.2–271.7)	29.82 (2.2–187.6)	0.195***
MDA, median (min–max)	4.56 (3.86–6.92)	4.42 (1.46–17.95)	0.023***
Total cholesterol, median (min-max)	172.12 (118.87–381.70)	167.49 (106.42–271.19)	0.177***
Triglyceride, median (min–max)	90.26(27.78–392.57)	131.41 (30.28–637.55)	0.229***
LDL, median (min–max)	105.30 (56.0–258.13)	98.36 (0–194.38)	0.117***
VLDL, median (min–max)	18.0 (6.0–79.0)	26.50 (6–128)	0.246***
HDL, mean ± SD	48.79 ± 12.58	41.83 ± 9.31	0.004**
CoQ10/T.chol, median (min-max)	0.17 (0.031–1.63)	0.18 (0.014–1.31)	0.433***

*P value was determined by chi-Square test **P value determined by Independent samples t-test ***P value was determined Mann–Whitney U test

Number of verruca lesions was significantly higher in patients with genital warts compared to patients without genital warts (P = 0.029). However, the duration of verruca, number of lesions, zinc, MDA, coenzyme Q10 levels, lipid profile as well as CoQ10/total cholesterol ratio were similar in patients with genital and patients with nongenital warts (Table 2).

**Table 2:** Descriptives, clinical and laboratory characteristics of the patients with genital and nongenital verruca vulgaris.

	Patients with genital verruca (n: 18)	Patients with nongenital verruca (n: 31)	P
Sex Female, n (%) Male, n (%)	5 (27.8%) 13 (72.2%)	23 (74.2%) 8 (25.8 %)	0.002*
Age, mean ± SD (median)	34.50 (18–64)	25 (18–65)	0.092**
Duration of verruca (month), median (min–max)	3.00 (1.00–120)	12.00 (1.00–120)	0.103**
Number of verrucas, median (min–max)	10 (1–100)	4.00(1.00–23.00)	0.029**
Treatment Cryotherapy, n (%) Others (imiquimod, salicylic acid and 5-fluorouracil) n (%)	15 (83.3%) 3 (16.7%)	28 (90.3%) 3 (9.7%)	0.479*
Zinc, median (min–max)	80.34 (61.3–113.9)	77. 59 ± 11.94 (76.71)	0.455**
Co enzyme Q, median (min–max)	37.73 (8.2–208.5)	42.01 (8.60–271.70)	0.455**
MDA, median (min–max)	4.65(4.21–6.84-6.92)	4.48 (3.86–6.92)	0.437**
Total cholesterol, median (min–max)	178.81 (118.87–329.36)	172.12 (128.71–381.70)	0.917**
Triglyceride, median (min–max)	125.81(37.85–283.99)	86.36 (27.78–392.57)	0.213**
LDL, median (min–max)	109.97(56.0–255.91)	105.30 (69.87–258.13)	0.772**
VLDL, median (min–max)	25.50 (8.0–57.0)	17.00 (6.00–79.00)	0.249**
HDL, median (min–max)	42.29 (28.00–70.13)	50.04 (30.62–83.08)	0.237**
CoQ10/T.chol, median (min–max)	0.162(0.031–1.57)	0.22(0.40–1.63)	0.520**

*P value was determined by chi-Square test **P value was determined Mann–Whitney U test

Age and LDL levels were found to be negatively correlated with coenzyme Q10 levels. Total cholesterol and Coenzyme Q10/total cholesterol ratio were positively correlated with the coenzyme Q10 levels. Number of warts were positively correlated with zinc levels (Table 3).

**Table 3:** Correlation coefficients between coenzyme, zinc and age, number of verrucas (Spearman).

	Coenzyme Q (r)	Zinc (r)	MDA (r)	Age (r)
Age	–.402**	.016	.239	≈1
Number of verrucas	–.103	.297*	.0.008	–.032
Total cholesterol	.377**	.213	.450**	–.496***
Triglyceride	–.213	.058	.364**	.274
LDL	–.428**	.216	.388**	.571***
VLDL	–.218	.055	.366*	.287*
HDL	.204	.087	–.014	–.183
CoQ10/T.chol	.960***	.168	–.254	–.472**
Number of verrucas	–.103	.297*	.008	–.032

*P < 0.05 **P < 0.01 ***P < 0.001

## 4. Discussion 

In this study, coenzyme Q10 and MDA levels were found to be higher in patients with verruca vulgaris while the serum zinc levels were significantly lower. This suggests that the presence of underlying oxidative stress in verruca vulgaris patients may affect the immunological response of these patients.

Zinc is an essential element that plays an important role in many biological processes in addition to its antioxidant effect [11,12]. Studies have shown that the antioxidant effect of zinc is achieved by inducing the activity of Metallothionein 5 (MT5). Metallothioneins (MT) are cellular proteins rich in zinc, copper and cysteine. These proteins are expressed in various tissues and protect against oxidative stress by inducing apoptosis. The antioxidant effect of MTs is mainly due to the presence of zinc in its structure. MTs act as free oxygen radical scavengers that inhibit DNA damage and lipid peroxidation. The sulfhydryl groups in MTs react with hydroxyl radicals and zinc is released to the environment. This results in a 300-fold greater binding of the sulfhydryl groups to free oxygen radicals, thereby reducing oxidative stress. Consequently, oxidative stress increases with zinc deficiency [11,13].

Clinical studies have indicated that zinc sulphate has therapeutic value. It was reported that intralesional application of 2% zinc sulphate in patients with verruca was efficacious and suggested that zinc could be among the treatment options [3,14]. In another study, oral zinc sulphate was started at 10 mg/kg dose in treatment resistant verruca patients; zinc was found to be highly effective in the treatment of resistant verruca compared to the control group [12]. Seareh et al. [15] reported that the combination of zinc sulphate with conventional therapies was effective as a treatment option and could reduce the relapse rate. In a randomized, double-blind, placebo-controlled study zinc sulphate was administered to 83 patients in addition to cryotherapy; however, no significant difference was observed in the outcome when compared to the placebo control group [16]. Raza et al. [17] reported low serum zinc levels in persistent verruca vulgaris patients with more than 10 warts and disease duration of more than 6 months and suggested that zinc deficiency might be involved in the resistant cases. In the current study, patients with verruca vulgaris were found to have significantly decreased serum zinc levels compared to the control group. However, no correlation was found between the location of the lesions (genital versus nongenital), duration of the disease, and serum zinc levels. These data support the role of zinc deficiency and resulting immunodeficiency in the etiopathogenesis of the disease rather than the duration of the disease. Interestingly, we observed a significant positive correlation between the serum zinc levels and number of warts. It is possible that a dysregulation in the transition of zinc from the serum to the tissue and thereby reduced zinc levels in the tissue could result in impaired antioxidant and immune functions leading to an increase in the number of lesions.

Reactive oxygen radicals are produced during normal tissue metabolism that are destroyed by an endogenous antioxidant system. If the antioxidant mechanism is perturbed, the resulting ROS can cause deterioration of cell functions due to the damage of lipids, DNA and proteins. ROS is also known to suppress T cell signalling, activation and proliferation [18].

The relationship between verruca and oxidative stress is well-established [5,19]. A number of immunological factors can be affected by oxidative stress; which, in turn can further exacerbate oxidative stress. MDA is a marker of oxidative stress indicating ROS induced lipid peroxidation. Arıcan et al. [5] examined that levels of MDA and the antioxidant enzymes catalase (CAT), and superoxide dismutase (SOD) in lesion and nonlesion skin samples of 36 patients with verruca vulgaris. The skin samples obtained from verruca lesions were found to have higher levels of MDA and lower levels of SOD compared to the nonlesion areas. These authors concluded that oxidative stress may play a role in the etiopathogenesis of verruca vulgaris. On the other hand, Çokluk et al. [19] evaluated serum CAT, MDA, paraoxonase and GSH-Px levels in 32 patients with genital warts and reported no significant differences with the control group. 

In the current study, corroborating the data reported by Arıcan et al., we observed significantly higher serum MDA levels in the verruca patients compared to the control group. Increased oxidative stress in these patients was likely to compromise not only the immune response, but also the affect the inflammatory process.

Coenzyme Q10 is found in all tissues in humans and plays a central role in the mitochondrial respiratory system in the synthesis of ATP. Coenzyme Q10 also acts as an antioxidant by interacting with oxygen-induced radicals, thereby preventing the onset of lipid peroxidation and damage of biomolecules. Coenzyme Q10 has also been shown to play a role in cell membrane stability, cell signalling, gene expression, cell growth, and apoptosis [8,20]. In addition, coenzyme Q10 is known to protect membrane phospholipids and low-density lipoproteins from peroxidation (20). A balance between oxidant and antioxidant levels was thought to contribute to the spontaneous regression of verruca lesions [21].

Coenzyme Q10 is transported with cholesterol and lipoproteins and its levels can therefore be correlated with plasma cholesterol concentration. So, the coenzyme Q10 to total cholesterol ratio has also been reported in the literature [22]. In the current study, both coenzyme Q10 and coenzyme Q10/cholesterol ratio were evaluated. Coenzyme Q10 level was high in the patient group compared to the control group. This suggests the likely presence of a feedback mechanism whereby enhanced lipid peroxidation and increased levels of MDA could lead to a greater requirement of antioxidant functions and higher amounts of coenzyme Q10. It also suggests that this molecule, which is affected by lipid parameters, may also affect other currently unknown regulatory mechanisms. In addition, no significant difference was observed in the coenzyme Q10 levels, coenzyme Q10/total cholesterol ratio and lipid profiles between the patient and control groups and between the genital and nongenital patient groups. Total cholesterol levels were found to be higher in the patient group compared to controls; however, this difference was not statistically significant. This suggests that oxidative stress is more likely to affect the pathogenesis of verruca vulgaris rather than its location. Moreover, although the coenzyme Q10 levels were found to be higher in the patient group, these patients also showed higher total cholesterol levels than the control groups, which resulted in no significant change in the coenzyme Q10/total cholesterol ratio. 

The low patient number and the fact that the markers were not simultaneously evaluated in the serum and tissues are the limiting aspects of the current study.

In conclusion, the serum levels of zinc in verruca vulgaris patients were lower compared to the healthy control group; additionally, this decrease was significant. Reduced serum zinc and increase of oxidative stress in verruca vulgaris may be a factor responsible for the development of verruca vulgaris. Thus, more studies with bigger sample sizes are needed to better evaluate the relationship between oxidative stress mechanisms and the immune system in the etiopathogenesis of verruca vulgaris.

## Informed consent

Informed consent was obtained from all participants before the study was commenced. The ethical approval for this study was obtained from the Ethics Committee of Süleyman Demirel University, Faculty of Medicine (number: 119).

## References

[ref1] (2018). Penile warts: an update on their evaluation and management. Drugs in Context.

[ref2] (2002). Human papillomavirus molecular biology and pathogenesis. Journal of the European Academy of Dermatology and Venereology.

[ref3] (2016). The Clinical Effectiveness of Intralesional Injection of 2% Zinc Sulfate Solution in the Treatment of Common Warts. Scientifica.

[ref4] (2017). Zinc in Infection and Inflammation. Nutrients.

[ref5] (2013). Status of oxidative stress on lesional skin surface of plantar warts. Journal of the European Academy of Dermatology and Venereology.

[ref6] (u2ca5). Coenzyme Q10 and immunity: A case report and new implications for treatment of recurrent infections in metabolic diseases. Clinical immunology.

[ref7] (2014). Coenzyme Q10 supplementation for the primary prevention of cardiovascular disease. The Cochrane database of systematic reviews.

[ref8] (2017). The effects of coenzyme Q10 supplementation on glucose metabolism and lipid profiles in women with polycystic ovary syndrome: a randomized, double-blind, placebo-controlled trial. Clinical Endocrinology.

[ref9] (1990). Malondialdehyde determinationas index of lipid peroxidation. Methods in Enzymology.

[ref10] (1979). Direct measurement of zinc in plasma by atomic absorption spectroscopy. Clinical Chemistry.

[ref11] (2019). Zinc Deficiency and Arsenic Exposure Can Act Both Independently or Cooperatively to Affect Zinc Status, Oxidative Stress, and Inflammatory Response. Biological Trace Element Research.

[ref12] (2002). Oral zinc sulphate in the treatment of recalcitrant viral warts: randomized placebo-controlled clinical trial. British Journal of Dermatology.

[ref13] (2018). The zinc-metallothionein redox system reduces oxidative stress in retinal pigmnet epithelial cells. Nutrients.

[ref14] (2018). Intralesional 2% zinc sulfate solution for plane warts: A case report. Dermatologic Therapy.

[ref15] (2014). Efficacy of combination therapy of oral zinc sulfate with imiquimod, podophyllin or cryotherapy in the treatment of vulvar warts. The Journal of Obstetrics and Gynaecology Research.

[ref16] (2017). Cryotherapy plus oral zinc sulfate versus cryotherapy plus plecabo to treat common warts: A double blind, randomized, plecabo-controlled trial. International Journal of Women’s Dermatology.

[ref17] (2009). Evaluation of oral zinc sulfate effect on recalcitran multiple viral warts: A randomized plecabo-controlled clinical trial. Journal of the American Academy of Dermatology.

[ref18] (2007). Oxidative stress modulation and T cell activation. Experimental Gerontology.

[ref19] (2015). Determining oxidant and antioxidant status in patients with genital warts. Redox Report: Communications in Free Radical Research.

[ref20] (u2c9b). Correlations between oxidative DNA damage, oxidative stress and coenzyme Q10 in patients with coronary artery disease. International Journal of Medical Sciences.

[ref21] (2012). Coenzyme Q(10), vitamin E, selenium, and methionine in the treatment of chronic recurrent viral mucocutaneous infections. Nutrition.

[ref22] (2000). Serum levels of coenzyme Q10 in patients with Alzheimer’s disease. Journal of Neural Transmission.

